# PyNCS: a microkernel for high-level definition and configuration of neuromorphic electronic systems

**DOI:** 10.3389/fninf.2014.00073

**Published:** 2014-08-29

**Authors:** Fabio Stefanini, Emre O. Neftci, Sadique Sheik, Giacomo Indiveri

**Affiliations:** ^1^Department of Information Technology and Electrical Engineering, Institute of Neuroinformatics, University of Zurich and ETH ZurichZurich, Switzerland; ^2^Department of Bioengineering, Institute for Neural Computation, University of California at San DiegoLa Jolla, CA, USA

**Keywords:** neuromorphic systems, spiking neural network, AER, NHML, VLSI, Python

## Abstract

Neuromorphic hardware offers an electronic substrate for the realization of asynchronous event-based sensory-motor systems and large-scale spiking neural network architectures. In order to characterize these systems, configure them, and carry out modeling experiments, it is often necessary to interface them to workstations. The software used for this purpose typically consists of a large monolithic block of code which is highly specific to the hardware setup used. While this approach can lead to highly integrated hardware/software systems, it hampers the development of modular and reconfigurable infrastructures thus preventing a rapid evolution of such systems. To alleviate this problem, we propose PyNCS, an open-source front-end for the definition of neural network models that is interfaced to the hardware through a set of Python Application Programming Interfaces (APIs). The design of PyNCS promotes modularity, portability and expandability and separates implementation from hardware description. The high-level front-end that comes with PyNCS includes tools to define neural network models as well as to create, monitor and analyze spiking data. Here we report the design philosophy behind the PyNCS framework and describe its implementation. We demonstrate its functionality with two representative case studies, one using an event-based neuromorphic vision sensor, and one using a set of multi-neuron devices for carrying out a cognitive decision-making task involving state-dependent computation. PyNCS, already applicable to a wide range of existing spike-based neuromorphic setups, will accelerate the development of hybrid software/hardware neuromorphic systems, thanks to its code flexibility. The code is open-source and available online at https://github.com/inincs/pyNCS.

## 1. Introduction

For over two decades, the Neuromorphic Engineering community has been developing the technology and methods for constructing micro-electronic circuits and devices that directly emulate the principles of computation used by the nervous system. These circuits and devices are aimed at building brain-inspired sensory-motor hardware systems that can implement neural computational models and interact with the environment in real-time (Mead, 1989; Chicca et al., 2014). Several examples have been demonstrated, ranging from autonomous reactive sensory-motor systems (Serrano-Gotarredona et al., 2009), to associative networks and learning systems (Mitra et al., 2009; Giulioni et al., 2012), to hybrid analog/digital systems for feature extraction and sensor fusion (Liu and Delbruck, 2010; O'Connor et al., 2013). More recently, several Neuromorphic *Computing* examples have been demonstrated, with hardware neural processing systems comprising large-scale re-configurable networks of spiking neurons, for both basic research goals (e.g., for understanding the principles of neural computation), and applied research goals (e.g., for applying alternative non-von Neumann computing paradigms to data mining or pattern recognition tasks) (Silver et al., 2007; Schemmel et al., 2010; Yu et al., 2012; Brain-Corp-Technology, 2013; IBM-Cognitive-Computing, 2013; Samsung-GRO, 2013). A recent Proc. of the IEEE Special Issue^1^ highlights the evolution of the field in the last 20 years (see for example Benjamin et al., 2014; Furber et al., 2014).

A common approach in building such large-scale spiking neuron hardware systems is to integrate multi-core or multi-chip re-configurable architectures. These architectures are typically characterized by mixed analog/digital processing within the neural cores and asynchronous digital communication across core/chip boundaries. While the details of the synapse and neuron circuits in the neural cores can vary significantly across the different types of neuromorphic devices being proposed (Indiveri et al., 2011), most of them share a common communication protocol, based on the Address Event Representation (AER) (Mahowald, 1992; Lazzaro et al., 1993; Boahen, 1998; Deiss et al., 1998). In this representation each computational element or node (e.g., a neuron or a synapse) is assigned an address. When a spiking element generates an event, its address is instantaneously put on a digital bus using asynchronous logic. Input and output signals are therefore represented as Address Events (AEs), and are typically produced and transmitted in real-time (i.e., time represents itself). Address-events can be tagged with additional “payload” information (e.g., a time-stamp for data logging, a list of destination addresses, a synaptic weight value, etc.). The digital nature of these events makes them ideal for routing and control using digital programmable hardware. With a large number of neuromorphic engineers adopting the AER, this representation has become the *de facto* standard for implementing communication protocols in neuromorphic systems. In turn, the adoption of this standard has contributed to rapid development of a large variety of re-configurable, modular and expandable multi-chip systems (Chicca et al., 2007; Imam et al., 2012; Patterson et al., 2012; Yu et al., 2012). Unfortunately, the definition of a common standard for signal transmission in neuromorphic systems has not translated to a common software infrastructure for accessing these systems. Every new hardware development effort typically leads to the development of new *ad-hoc* software to support custom interfaces, drivers, and high-level software modules for carrying out experiments with that specific hardware. This situation is not ideal since it hampers the possibility of integrating components from different neuromorphic approaches to create sophisticated multi-chip systems.

In this paper we present a Python software framework that exploits the common features and attributes of neuromorphic electronic systems to control and carry out experiments with them. It provides a front-end that facilitates the replication of neural network models onto a heterogeneous set of neuromorphic hardware platforms communicating with each other through AER. In PyNCS, the low-level functionalities are separated into different independent and reconfigurable blocks imported into the core module. PyNCS manages the intercommunication within these blocks and facilitates the functionalities needed for the neural network definition, the configuration of the neuromorphic hardware and the experimentation with it. Thus, this approach offers a flexible way to combine devices into a unified software *ecosystem* and facilitates hardware and software development. As this software aims to design and construct neuromorphic agents endowed with high-level “cognitive” computational abilities (Neftci et al., 2013), we named it “PyNCS” (Python framework for Neuromorphic Cognitive Systems). The use of the Python programming language for PyNCS encourages code reuse, e.g., through class inheritance, a key property for the modularity of our software. Modules and wrappers permit a convenient extension to programs written in other languages such as C and C++, which enables third-party drivers and libraries to be easily integrated in the system. As Python is an interpreted language, PyNCS permits easy scripting and debugging with benefits for software development and experimentation with the models. Furthermore, the large repertoire of libraries available in Python can be readily included in the PyNCS modules for optimizing the code that defines and runs the experiments, the data analysis and its visualization.

In the next sections we present the general structure of PyNCS and compare it to related approaches described in the literature. In particular, in Section 2 we present the classes of PyNCS that are used to control the setup, handle address events and define the neural populations and their connectivity profiles, in Section 3 we describe the APIs that are integrated in PyNCS to interact with the low-level drivers and in Section 4 we show two representative application examples that demonstrate the flexibility and usefulness of our software.

### 1.1. Software ecosystems for neuromorphic electronic computing platforms

Several software platforms for controlling neuromorphic systems have already been developed. For example, the jAER software (jAER, 2006) is an open-source project, written in Java, that allows *soft* real-time processing of event-based data from spiking neuromorphic chips. Initially developed to process signals produced by the Dynamic Vision Sensor (DVS) (Lichtsteiner and Delbruck, 2005), it contains a large library of modules (or “filters”) for applying algorithms to streams of spikes, e.g., for the computation of the optical flow. Expanding the software to support new hardware devices often requires direct programming of Java classes with the low-level instructions needed for parameter controls and AER communication. To date, jAER doesn't include modules for generating and sending AER events to neuromorphic chips, or for defining and configuring neural networks.

A widely adopted software package for experimenting with neural networks is PyNN (Davison et al., 2009). PyNN, initially designed as a Python simulator-independent neural network definition framework, has also been used to map neural network architectures onto different neuromorphic hardware platforms. The PyNN front-end permits the definition of the neural network in terms of neuronal populations, connections and parameters. To translate these definitions into hardware configuration and to allow data communication with different hardware architectures and systems, a dedicated hardware abstraction layer needs to be written for each system considered. Typical approaches for the design of the hardware abstraction layer consist in writing the required code in the desired programming language (e.g., C or C++), or in integrating it into more high-level programming frameworks, such as Python or MATLAB (Brüderle et al., 2007; Giulioni et al., 2012; Wijekoon and Dudek, 2012; Brink et al., 2013; Painkras et al., 2013).

The novel/unconventional nature of neuromorphic systems (e.g., compared to conventional von Neumann computational systems), requires a novel approach to system design. At the same time, the assortment of hardware platforms being developed in both academic and industrial research areas is growing fast. The *ad-hoc* solutions described above are only a part of the large spectrum of dedicated software which is used to configure and interface the electronic hardware systems. Ultimately, these low-level software infrastructures should facilitate the applicability of new programming paradigms through high-level front-ends. One recent attempt for the construction of such exhaustive ecosystem has been recently announced by IBM researchers (Amir et al., 2013). It relies on an abstraction layer composed of *Corelets* to represent networks of “neurosynaptic cores” and a novel programming language for composing them and obtain the desired functionalities. The Corelet language can be seen as an exhaustive neural network front-end, spanning from the specification of neuron-to-neuron connections to high-level descriptions of computational modules, such as recurrent networks or Winner–Take–Alls (WTAs), and is embedded in a complete development platform called “Corelet Laboratory.” However, as opposed to simulator or hardware independent solutions such as PyNN, the Corelet software is tied exclusively to the specifc hardware architecture of IBM's chips and so its applicability is restricted to those particular platforms.

In general, all the available solutions, including IBM's Corelet Laboratory, rely on a low-level abstraction layer for interfacing the custom hardware to the user front-end, a middle-layer for translating the model parameters into hardware parameters, and a high-level front-end with APIs for the definition of the neural network and data analysis. The solution we propose complies with these general principles and integrates all different modules into a unified reconfigurable framework.

**Figure 1:**
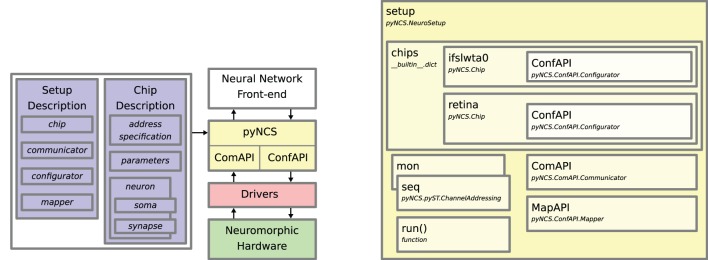
**Overview of PyNCS**. **Left:** The “Setup description” and “Chip description” blocks represent XML files that provide the specifications of the hardware. A user-written Python script describes the neural network model and interacts with PyNCS to send data to and gather data from the system. Communication with the low-level drivers and the neuromorphic hardware is mediated by the communication and configuration API modules. These modules implement the interaction with the custom drivers, which can take the form of executables or code libraries. The arrows represent the data flow in the system. **Right:** The main components of the Setup class. The setup object serves as the gateway for performing all the operations involving the hardware. It contains the objects necessary for accessing the chips' biases and parameters (in this example, two chips ifslwta0 and retina), the objects that translate raw AER addresses into human readable ones (mon, for monitoring events and seq for sequencing them) and the API modules for configuring the network topology (pyNCS.ConfAPI.Mapper) and for mediating the spike-event communication (pyNCS.ComAPI.Communicator). Examples of hardware specification XML files are provided in the supplementary material.

An overview of PyNCS and the relationships between its components and the hardware is illustrated in Figure 1. The “Setup Description” and “Chip Description” files encapsulate all the information that characterizes the hardware setup such as the number of chips, the number of neurons in each chip, their parameters, but also identifies which libraries (drivers) to load in order to access the hardware (implementation details in the Supplementary Material). These files, along with a description of the neural network and the experiment, are specified in a user-written Python script that uses the PyNCS front-end to send data to and gather data from the neuromorphic system. A typical script consists of the following steps:

1. A setup definition part where the system initializes internal elements needed to communicate with the hardware for monitoring or configuration, e.g., enabling or probing communication with the drivers;
2. A population definition part where neuronal populations are declared and hardware resources are automatically assigned according to the script commands, i.e., corresponding to the neuron elements chosen for the experiment;
3. A network definition part where look-up tables corresponding to the desired network topology are generated;
4. A configuration part where circuit parameters are set, e.g., neuronal or synaptic time constants
5. A “run” part where the network is applied on the hardware and activity is continuously monitored by dedicated objects;
6. A final part with the desired storage, analysis, and plotting of the data.

When such script is run, PyNCS automatically generates the necessary elements to access the hardware resources. The interface between the high-level neural network definitions and the neuromorphic hardware is mediated via special API modules (described in detail in Section 3) which integrate the hardware drivers into the PyNCS framework. With the use of modules as common blocks of a stereotype neuromorphic systems, PyNCS can be used as a common ground for integrating various kinds of neuromorphic devices and for running experiments with spike-based hardware both in continuous/interactive and batch/off-line mode.

## 2. The neuromorphic setup as a spike-event transceiver

The main objective of PyNCS is to provide a common framework for the integration of different types of neuromorphic devices. This objective is realized with the choice of describing the neuromorphic chips as re-configurable address-event transceivers, i.e., they produce and/or consume AER events and have some degree of reconfigurability. We argue that this assumption is general since it covers the major AER-based hardware platforms developed in the community. In the development of PyNCS we thus aimed at finding the most general definition of a hardware setup in terms of address specifications, address mappings for realizing the neural network connectivity and parameters for permitting the correct functioning of the hardware. In other words, the structure of PyNCS is defined based on the minimum common denominator of spike-based neuromorphic setups but also provides access to all the specific functionalities provided by the particular devices adopted. Because of its nature, we identified several commonalities with the concept of a “kernel” in traditional computer architectures, which serves as a manager for input/output requests between applications and processing units. This mindset has been kept throughout the development of every part of the software and constitutes the philosophical ground on which it is built. More details on the above aspects and on the differences with existing solutions is provided in the Discussion.

The modularity of PyNCS is realized by separating the driver implementation from their configuration. The configuration specifies the parameters that drivers need in order to access hardware data, so that the same implementation can be used on different platforms. We decided to adopt a general descriptive model for the hardware systems that is (1) human readable to promote sharing, (2) flexible enough to cover the peculiarities of the hardware systems while allowing the existence of templates and (3) easily parsed by the software. These requirements are common in many other fields of computer science thus we opted for eXtensible Mark-up Language (XML) description files parsed by dedicated API modules. In this Section we describe in details the main classes of PyNCS and their use of the XML files.

### 2.1. The neuromorphic hardware mark-up language

In PyNCS, a dedicated Python class, the pyNCS.Chip class, is an abstraction layer used to control and communicate with the transceiver chips. The single chip specifications, such as the AER encoding and decoding parameters, are listed in dedicated Chip Description XML files, or simply *chip files*. Because of the generality of the structure on which these files are based, we defined a new dedicated mark-up named Neuromorphic Hardware Markup Language (NHML). In this description the chip is abstracted as one or multiple arrays of “neuron” elements, each having a “soma” block and, if present, “synapse” blocks. The soma block specifies the possible address events that can be generated by the spiking activity of the chip (*source* addresses), while synapse blocks specify the addresses that can be targeted from external sources or other neuromorphic chips (*destination* addresses). In order for the system to correctly encode and decode these addresses, an “address specification” block is provided in the NHML description with the codes that PyNCS uses for address translation. Finally, the NHML file contains a list of block and circuit parameters, with extra information fields for their typical values and with the information needed to access them, (e.g., chip pin numbers, addresses, etc.). Examples of NHML files are reported in the Supplementary Material.

### 2.2. The definition of a neuromorphic ecosystem: the multi-chip setup

In order to correctly encode and decode the address-events, PyNCS makes use of *address specification* codes. The code, which depends on the particular implementation of the neuromorphic chip, is parsed from the respective chip file. These addresses are essential not only to visualize the spiking activity and deliver synthetically generated spikes to the chips but also to construct the topology of the neural network. To automate these tasks at run time, a set of encoding/decoding functions are dynamically generated when the setup is initialized. These functions convert the addresses of any neuron or synapse (or any other addressable element) in the setup to a more convenient representation. Namely, they convert *physical* addresses encoded as integers into tuples of “Human Readable (HR)” coordinates which are the coordinates of the corresponding neuron (or the synapse) on the chip. Since the conversion between physical and HR addresses can be onerous at runtime, PyNCS makes extensive use of hash-tables. Hash-tables significantly speed-up the encoding/decoding of the events by implementing a dictionary of addresses easily created and accessed using Python built-ins. When rapid event translation is not needed, such as in batch-processing mode, the raw AER data is not translated and the communication throughput is limited only by the driver and the hardware. More information on the latency can be found in the Discussion, while implementation details regarding the address specification files and their use in address translations are provided in the Supplementary Material.

The information contained in the setup and chip description files is used by PyNCS to create an object of the pyNCS.NeuroSetup class. This object allows complete control of the hardware interfaces through the imported APIs. Figure 1 (right) shows an example of a NeuroSetup object and its main components for a system that consists of two neuromorphic chips. A typical setup object contains a Python dictionary of pyNCS.Chip objects, which can be used to configure the chip parameters through the ConfAPI.Configurator API. The setup object can send (*sequence*) and receive (*monitor*) spike events from/to the system using the methods implemented in the ComAPI.Communicator API. These events are translated into the different forms, physical or HR, using the address specifications and the functions contained in two pyST.ChannelAddressing objects.

**Figure 2:**
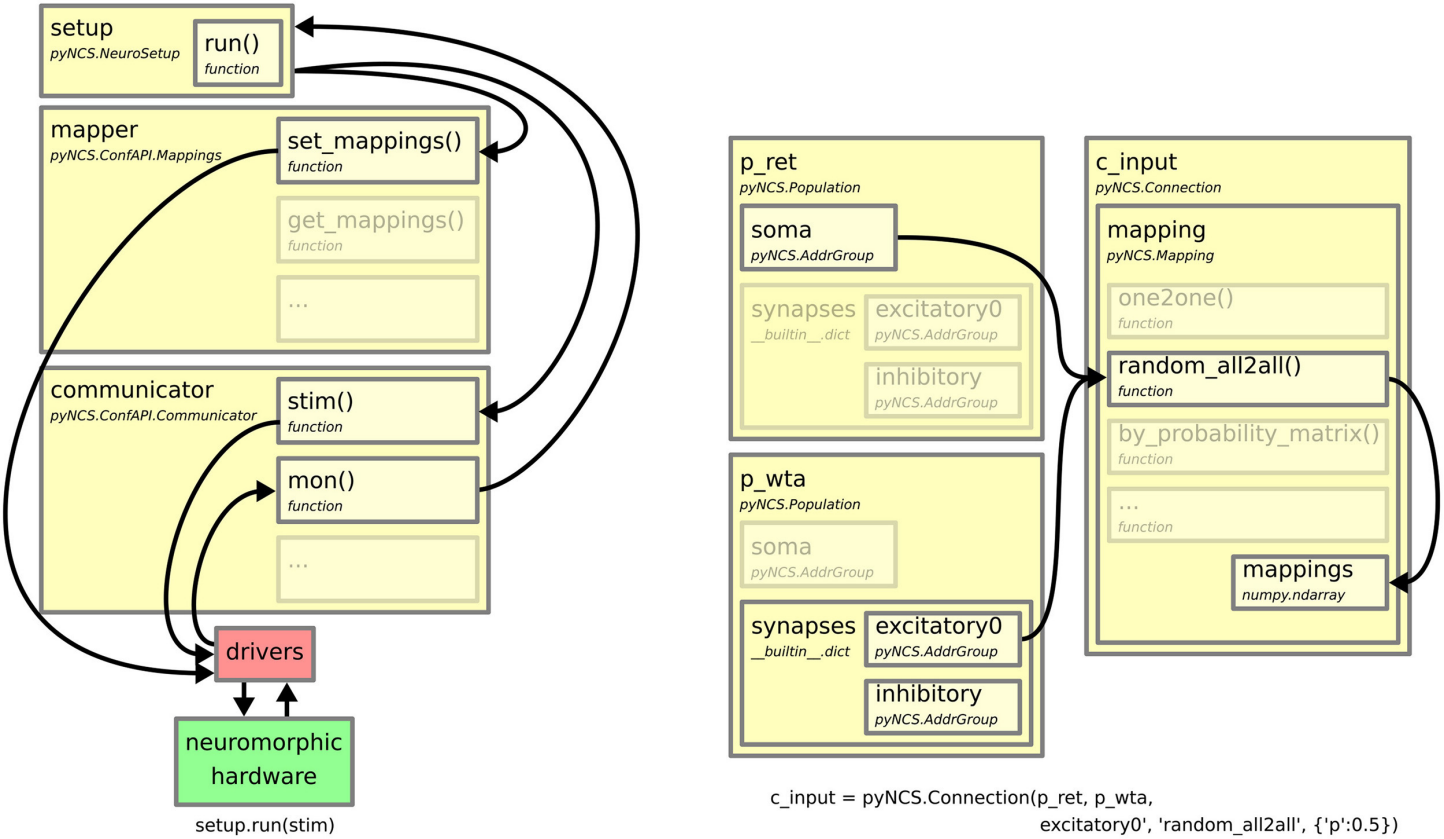
**Left:** Dataflow of an experiment run. The setup.run function configures the routers according to the network topology, defined as a map between source addresses and destination addresses, and sends the stimulus to the chips calling methods of the configurator and communicator APIs. These APIs implement calls to the custom drivers for communicate with the hardware. The communicator API monitors the spiking activity which is then collected by the run function. The arrows represent the data flow. **Right:** Constructing a connection between two populations, the p_ret and the p_wta, which have been previously defined. Upon creation, the connection class creates a look-up table between the address group of the pre-synaptic neurons' soma addresses and the excitatory synapses of the post-synaptic neuron. In this example, the connections are random with a 50% probability that a neuron in p_ret connects with a neuron in p_wta. The list of connections is stored in a look-up table, mapping, which is transfered to the hardware when the “run” function is called. The arrows in the figure show the data flow between the different PyNCS classes.through setup.run.

While the NeuroSetup class has been conceived with the primary intent of providing full online interaction with the neuromorphic system, a dedicated run function has been also included in its implementation for batch-processing. This function executes in one shot all the operations that are needed to configure the hardware resources to match the desired neural network model, activate the communication to sequence and monitor the spiking data and store the results. Figure 2 (left) shows the data flow during the execution of this function and the interaction between the APIs that realizes the batch processing.

### 2.3. From abstract addresses to neuronal populations

The PyNCS front-end provides the user with a set of tools to map a given neural network model onto the specific hardware. To this end, we implemented a set of user-friendly Python classes which resembles the ones found in most neural simulators and usually consist of populations of neurons, connections between them, and monitors (Brette et al., 2007). In addition, the APIs in PyNCS provide easy access to low-level drivers through the dedicated pyNCS.NeuroSetup class.

The class pyNCS.Population mirrors similar classes found in typical neural network simulators, such as the NeuronGroups class of the Brian neural-network simulator (Brette et al., 2007). It represents a group of neurons and their associated synapse circuits, i.e., the ones that are physically wired to them. In fact, the neurons in a population and their synapses are treated as two groups of addresses with a class pyNCS.AddrGroup, which inherits Numpy's structured arrays (http://www.numpy.org). Typical array operations have been remapped to operate on the Population object for easily pointing at subgroups within the population, to evaluate simple population properties, such as its size, or to create sub-populations.

The instantiation of a neural population in a simulator running on a traditional machine typically uses the number of desired neurons and their dynamic model as the only arguments to allocate enough memory for their operations. Parameters are then “attached” to this population and used by the simulator to modify the neurons' internal state at runtime.

Instead, in neuromorphic systems, neural parameters and even network behavior can depend on the exact location of the circuits. This variability can be caused by circuits mismatch, communication delays that affect the asynchronous communication, or the presence of faulty elements in the system. Thus, it is important to have control on the *placement* of neurons onto the hardware, which often involves dedicated algorithms or *neural network compilers* (see for example Brüderle et al., 2011; Cruz-Albrecht et al., 2013; Navaridas et al., 2013). In PyNCS, the modeler has direct control on the desired placement by being able to directly select the addresses of the neurons composing the desired population, if needed. For convenience, the Population class implements several simple placement methods, for example a linear method, i.e., a given number of addresses are selected as they are listed within the population array. The populate methods have access to the Setup object through the Population object, making it possible to include any arbitrary placing method. For example, the Setup object could store calibration parameters of each neuron element and the Population could be selected using this information for precise control on neuron parameters.

### 2.4. Definition and configuration of the network topology

In an AER communication system, the network topology is implemented by dedicated digital devices that route the address events generated by neurons or sensors to the corresponding destination synapses. The simplest router uses a Look-Up Table (LUT) stored in a memory to generate synapse events for every neuron event. We shall refer to such device as a *mapper*, since it assigns an exact correspondence between source addresses and destination addresses. While there exist more efficient representations for the network connectivity, for example in terms of memory occupation, the LUT is probably the most intuitive way of describing a connectivity profile to which any other description can always be converted. This choice resulted to be very convenient, e.g., for debugging or direct visualization and separates the problem of the definition of the network connectivity from the problem of configuration of the hardware to reproduce that connectivity, a function which is operated by the Configurator API (see 3.2).

In PyNCS, the connectivity between populations is generated by pyNCS.Connection classes through the connect methods. Several common methods for generating connectivity patterns are available in the current release of the software, such as one-to-one or all-to-all profiles. The LUT that correspond to the desired connectivity profiles are generated internally by appropriately selecting the addresses from the ones that are present in the Population objects. The address arrays consist of Python's structured arrays to facilitate appropriate filtering on soma and synapse addresses. Though generator functions for commonly used connectivity profiles are used, the user has also direct access to the arrays of addresses within a population. Hence, the user has the possibility to generate arbitrary connectivity profiles by explicit selection of the corresponding addresses. This possibility goes along the lines of the implementation philosophy of PyNCS, which aims at facilitating common operations with dedicated functionalities but also at allowing arbitrary configurability from the front-end. Figure 2 (right) shows an example of the creation of a connectivity profile between two excitatory populations with a 50% probability of each connection to exist.

### 2.5. Monitoring spiking activity

In order to conveniently display the spiking activity of the devices involved in the system PyNCS includes a dedicated “monitor” module. The main role of the monitors is to automatically elaborate meaningful representations of the spiking activity in the network, e.g., for plotting purposes. The pyNCS.Monitor class implements methods that are used to collect raw AER data and distribute it according to given populations or subpopulations. For this purpose, the monitors access all the informations needed for address translation that are provided by the setup class. In batch mode, the AER events are interpreted and eventually visualized only at the end of the batch process. The use of monitors helps in selecting only the populations of interest and obtain intuitive visualizations. We discuss performance issues, for example for real-time visualization, in Section 5.2. A usage examples of the monitors is provided in the Supplementary Material.

### 2.6. Sequencing recorded or synthetic spike trains

Spiking data are handled as lists of pairs of physical addresses and spike-times (see also Supplementary Material). The data can be recorded from an experiment using the monitoring functions of PyNCS, generated using routines that come as methods within the Population objects, or generated using numerical functions and then associated to the corresponding physical addresses before sequencing. Any population defined in the network can be used as a source of spike events, i.e., a spike time can be associated to any physical address available in the network. Importantly, while the user can also generate sophisticated spike train objects by careful selection of the desired physical addresses, the Population object can also handle address translations that are required to generate the spike trains from the HR addresses. In this way, PyNCS allows for sophisticated interactions between the elements in the network, which is needed for example for the introduction of precisely timed spike events. Notice that also physical destinations, such as synapses, can be used to generate spike-trains, e.g., for testing purposes. For example, spike-trains with synapse addresses could be directly sent from a host computer into the neuromorphic system without the need for mapping these events to target the corresponding physical synapses. Once the spike trains are generated or collected, they can be injected into the system at runtime or using the run function for batch processing, as we explain in more detail in the next section.

## 3. Modular architecture of PyNCS

The low-level drivers for communication and configuration are specific to each type of neuromorphic hardware. PyNCS makes use of Python's object oriented paradigm to efficiently handle this variety, by making use of API modules. These APIs consist of custom classes that manage the interaction with the hardware-specific drivers. Developers can use a base class, for example pyNCS.ConfAPI.Configurator, to implement the custom class and so take advantage of class inheritance. The base classes contain the necessary modules that PyNCS expects from the module, i.e., the *least common denominator* of each module. At the same time, the module can contain dedicated functions that permit access to particular functionalities of the system in use. Examples of such functions for a “configurator” API module are get_parameters and set_parameters. One way to implement the API is to use plain Python code and make calls to specific executables accessing the driver resources and so the device. A more direct way is to use wrappers or code binding to integrate the drivers into the Python code, using for example SWIG (http://www.swig.org/). The APIs cover the main modules needed to operate the neuromorphic system:

- a configuration module, to configure the analog and digital devices for proper functioning, e.g., for setting the parameters of the neural circuits;
- a communication module, for handling the AER communication with and between devices, e.g., to monitor the spiking activity.

For testing purposes, PyNCS is distributed with an API that interfaces with the Brian Neural simulator (pyNCS.api.BrianAPI) (Goodman and Brette, 2008).

### 3.1. Communication API

The communicator API, ComAPI.Communicator, mediates the communication of events between the PyNCS environment and the neuromorphic hardware. The primary function of the communicator API is to send (i.e., sequence) and receive (i.e., monitor) AER events to and from the AER bus. In addition to the send/receive functions, the base class of this API also defines a run method that enables batch event communication with the hardware.

The communicator API is used to separate the complexity of the communication of the events from the encoding and decoding of the addresses which take place internally in PyNCS. Consequently, the run function expects and returns physical addresses, in the form of Numpy arrays of integers. This makes it possible to easily integrate remote communication protocols (e.g., TCP/IP) into the API since the address events can be sent as packets of integers.

An additional useful functionality integrated in the communicator API is to enable the execution of arbitrary processes at the time of the ability activation of the communication with the setup. For example, for some experiments, it is necessary to precisely synchronize the sequencing and monitoring with an external event, such as the initiation of a visual stimulus. PyNCS makes use of Python's “context managers” to define such runtime contexts. These can be conveniently implemented using the decorator function contextmanager available from Python's built-in contextlib module, and passed as an argument to run. The module establishes the context by placing the call to the low-level driver functions under a with statement. For an example see Section 4.1.

### 3.2. Configuration API

The configurator API handles the control of low-level hardware parameters, e.g., programming digital/analog converters or on-chip bias generators used to set the parameters of the neural circuits. Parameters such as firing thresholds, neural leakage currents, synaptic integration time constants and so forth are controlled through the configurator API, which translates calls to functions such as set_parameter into driver calls. Several methods exist for configuring neuromorphic chip parameters to achieve a target functionality or to match those of a theoretical model (Russell et al., 2010; Brüderle et al., 2011; Neftci et al., 2011; Gao et al., 2012). While PyNCS does not address the issue of parameter translation directly, the configuration API offers a convenient interface to include these methods into the software ecosystem as separate routines. Additionally, the configurator API is intended to also handle any other aspect related to the proper functioning of the neuromorphic setup, e.g., the activation of some powering schemes or the proper setting of specific digital controls.

The configurator API also handles the configuration of the routers for implementing the neural network connectivity. This functionality is encapsulated in the mapper API sub-module. This module takes care of “compiling” the neural network definition into a configuration table for the routing of the AER events in the setup according to the available hardware resources. For example, the API can apply methods for converting the LUT representation of the connectivity into dedicated routing schemes, such as hierarchical, multi-cast, broadcasting or tag-based schemes (Northmore and Elias, 1998; Furber et al., 2006; Merolla et al., 2007; Joshi et al., 2010; Moradi and Indiveri, 2014) or for appending additional parameters such as delays or probabilistic spiking in the configuration of the routers.

## 4. Usage examples

In this Section we describe in detail the use of PyNCS with the neuromorphic system developed at the Institute of Neuroinformatics (INI) in Zurich. We report here two examples that demonstrate the basic capabilities of PyNCS for carrying out experiments requiring continuous interaction with the neural network emulation and local computations. However, since most of the experiments carried-out at INI during the last 5 years have used PyNCS, we refer the reader to the literature to gather more examples of the use of PyNCS for models involving rate-based computation but also precise spike-timing (Sheik et al., 2012; Chicca et al., 2014).

### 4.1. Interfacing a spiking neuromorphic chip with a silicon retina

Neuromorphic sensors emulate the functionality of biological sensors in analog/digital Very Large Scale Integration (VLSI) (Liu and Delbruck, 2010) and emulate some characteristics of the sensory pre-processing of the nervous system. These devices provide address-events in an asynchronous, event-based fashion and are thus ideal for interfacing neuromorphic spiking neural networks with the real-world. Here, we demonstrate the use of PyNCS in configuring and monitoring a setup with neuromorphic vision sensor connected to a multi-neuron chip. The multi-neuron chip implements a Soft Winner–Take–All (sWTA) by means of hard-wired connections. Neural networks implementing sWTAs have key features for information processing, such as signal restoration and amplification (Douglas et al., 1994), and thus are useful building blocks for synthesizing large scale models of cortical computation (Rutishauser and Douglas, 2009; Neftci et al., 2013; Chicca et al., 2014). In this toy experiment we will demonstrate the selective amplification property which permits the network to track the strongest of two visual stimuli.

The multi-neuron chip consists of a network of I&F neurons (Indiveri et al., 2006), with dynamic synapses (Bartolozzi et al., 2006). The chip contains a total of 124 excitatory neurons with hard-wired first, second and third nearest-neighbor excitatory connections and 4 inhibitory neurons. These inhibitory neurons receive excitatory connections from all the other neurons and inhibit them through inhibitory synapse circuits. The multi-neuron chip receives spikes from a DVS, often referred to as “silicon retina,” with 64 × 64 pixels (Lichtsteiner and Delbruck, 2005). Each pixel is sensitive to temporal contrast changes and emits AER events independently. Each column of the vision sensor sends its activity to one neuron in the sWTA, through an external digital mapper (Fasnacht and Indiveri, 2011). Notice that the connectivity is realized by means of both hard-wired and AER-routed connections, once again expressing the flexibility of PyNCS in managing the configurability of both conventional and specific parameters of spike-based neuromorphic systems.

**Figure 3:**
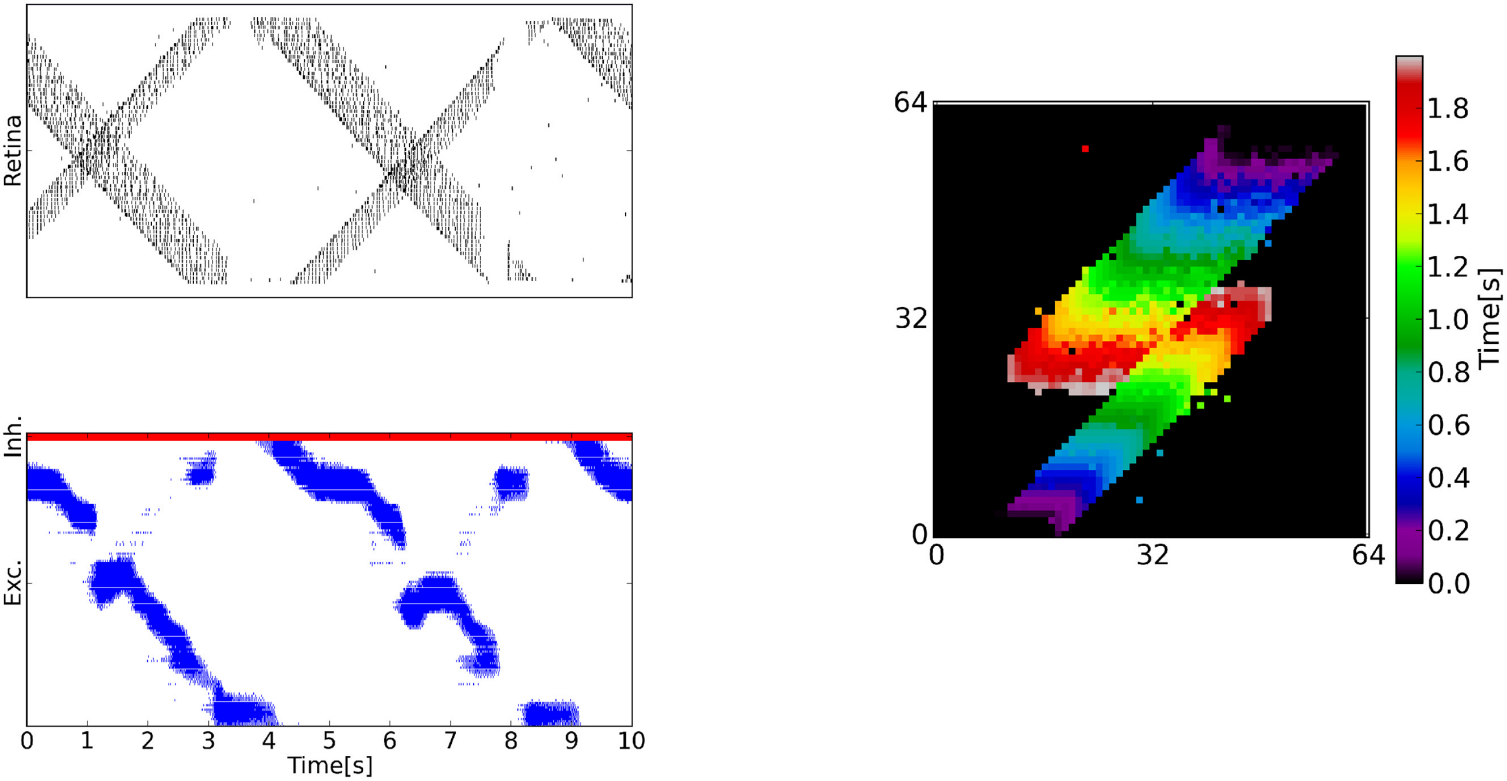
**A multi-neuron chip implementing the soft WTA function, interfaced with a silicon retina**. **Top left:** Raster plots of the retina's column–wise output activity. The pixel columns of the retina project on to the multi-neuron chip, such that each neuron received inputs from 2 columns. **Bottom Left:** Raster plot of the sWTA chip, where blue indicates the excitatory neuron events and red indicates inhibitory neuron events. The retina activity causes the winner-take-all to activate around the stronger stimulus most of the time, corresponding to the X-position of the longer bar. Occasionally, the sWTA activity jumps to the location of the shorter bar (e.g., around 3 s). **Right:** First 2 s of the retina output. The axes represent the X-Y coordinates of the events and the color encodes time. The stimulus consists of horizontal bars of different size moving in two opposite directions.

The silicon retina is placed in front of a standard LCD screen displaying a visual stimulus. The stimulus consists of two sliding bars of different size (see Figure 3) and has been produced using PyGame^2^. The sWTA selects the strongest input from the retina, which corresponds to the horizontal position of the largest bar in the moving scene, and tracks it throughout the movement in the scene.

We set up two neuromorphic systems, one controlling the silicon retina located at the Institute of Neuroinformatics in Zurich and one controlling the multi-neuron chip and the neural-network connectivity located at the Institute for Neural Computation in La Jolla. The spikes were transmitted using the TCP/IP protocol and a Python “socket,” a low-level networking interface widely adopted in computer science. The TCP/IP protocol was integrated in a dedicated API which was specified in the configuration files of the neuromorphic system. While the TCP/IP protocol does not guarantee real-time communication, the communication of the address-events over the network takes place in only one direction. As a result, the computation in the sWTA is slightly delayed with respect to the retinal output, but remains unaffected otherwise. The use of the TCP/IP protocol shows how existing protocols can be used in the ecosystem for specific functionalities, such as remote communication.

### 4.2. Synthesis of “soft state machines” for performing cognitive tasks

A hallmark of cognitive behavior is the ability of an agent to select an action based not only on specific external stimuli but also on their context (Dayan, 2008). To perform cognitive tasks, an agent must construct context-dependent sensori-motor mappings, requiring mechanisms for working memory, decision making and action selection. One possibility to solve such cognitive tasks in spiking neural networks is to synthesize SSMs (Neftci et al., 2013), which are state-machines implemented using networks of sWTA circuits.

Neftci et al. have proposed a systematic procedure to map a given Finite State Machines (FSMs) diagram into its analogous SSM implementation composed of silicon neurons, as follows. A recurrently connected sWTA network contains populations of neurons that are able to reliably maintain persistent activity and are in competition with each other by means of long range inhibitory connections. Because of the competition, only one population can be persistently active and so this network can maintain the state of the FSM. An additional “transition” sWTA network responds selectively to combinations of external stimuli and internal states and triggers state transitions through excitatory connections. An FSM is mapped on the spiking neural network by introducing sparse connections from the transition sWTA to the appropriate state population as specified by the state diagram (Rutishauser and Douglas, 2009).

**Figure 4:**
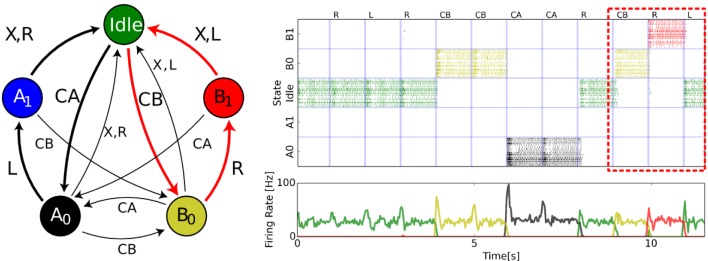
**Soft State Machine implemented in neuromorphic hardware**. **(Left):** State machine description of a cognitive task (Neftci et al., 2013). Each state is represented by a circle and each arrow is a possible transition given the annotated symbol. The symbol × reflects an “invalid” cue and resets the state to “Idle.” The spiking neural network architecture underlying the SSM is composed of three sWTA circuits: a state sWTA that maintains the state in persistent activity (32 neurons per state), a transition sWTA that mediates the transitions between states given the input symbols (16 neurons per symbol). The colored arrows in the state diagram indicate the path followed when the sequence *CB*, *R*, *L* is presented. **(Right):** State population activities recorded from a multi-chip neuromorphic setup. From top to bottom, raster and population firing rates of the state populations, raster and populations firing rates of the output populations. The labels situated above the top raster plot indicate the presented symbols. One valid sequence (*CB* followed by *R*,*L*) is detected at *t* = 12 s, as highlighted by the red box.

Figure 4 shows an example state-machine capable of detecting sequences of two symbols (*L, R*) or (*R, L*). The particular sequence to detect is specified by the symbols *CA* and *CB*. The transitions are defined by the FSM and are triggered by the arrival of each symbol. In the presented FSM, the internal states *A*_0_, *A*_1_, and *B*_0_, *B*_1_ not only represent the current symbol but also a task-relevant trace of the preceding symbols. The SSM starts from “Idle” and progresses through the states as a randomly generated sequence of symbols is presented. An additional symbol × reflects an “invalid” cue and resets the state to “Idle.”

The presented state machine was previously employed to detect the direction of motion of targets across a display with the use of spiking vision sensors (Neftci et al., 2013), and to solve a context-dependent delayed 2-back task (Dayan, 2008). The details and the code listing for configuring the SSM are provided in the Supplementary Material.

## 5. Discussion

With the increase of complexity of neuromorphic systems composed of spiking multi-neuron chips and sensors, the identification of a tool-chain (ecosystem) for the configuration of setups is a fundamental step for their rapid development. Python is an ideal programming language for this task for several reasons: (1) There are many possibilities for interfacing with existing code written in other programming languages such as C/C++, greatly reducing the effort in reusing existing hardware drivers; (2) It is object-oriented, with advantages for the definition of APIs and modularity of the software; (3) Its scripting capabilities combined with the scientific computing software, such as NumPy and SciPy, greatly facilitate experimentation and analysis for both continuous interaction and batch-processing; and (4) It is easy to learn for scientists and engineers who do not have extensive training in computer programming. The design of the PyNCS front-end resembles those of common neural simulators^3^, thus guaranteeing researchers an intuitive transition from scripting for software simulations to experimenting with hardware emulations. Here we discuss in detail some of the most important aspects of PyNCS.

### 5.1. Comparison with existing solutions

Perhaps the closest software framework to PyNCS in terms of functionality is PyNN (Davison et al., 2009) (see also Section 1.1). The key purpose of PyNN is to provide researchers with a unified front-end for neural network simulations, thus favoring reproducibility of results in computational neuroscience and allowing systematic comparisons between software simulators. The PyNN interface has been widely adopted due to its similarities with common neural network simulators and the possibility to easily port scripts to different simulators without coding efforts. More recently, PyNN has been used for neural network definition in experiments involving neuromorphic hardware. This has been achieved by implementing dedicated software modules, imported in PyNN, for configuring the parameters of neuromorphic hardware and for implementing the necessary translations of data and parameters between the high-level PyNN interface and the low-level drivers. For example, in Brink et al. (2013) the authors used MATLAB-based tools for defining the network topologies and parameters following the syntax of PyNN and then used PyNN as the front-end for the neural network definition. The low-level configuration functions were operated by custom MATLAB scripts that were already designed to provide access to the specific features of the neuromorphic system. Similarly, Manchester's SpiNNaker system, a digital, general purpose hardware platform for large-scale simulations of spiking neural networks (Painkras et al., 2013), is supported using this approach^4^. However, the increasing modularity of the hardware setups, their diversity, the complexity of the devices, their heterogeneity as well as the intensifying use of commercially available interfaces as building blocks of large hardware setups (e.g., USB interfaces, TCP/IP remote communication, FPGA-based routers) call for a similar modularity and flexibility in the software ecosystem. As PyNN was designed to provide a common front-end for neural network definitions across software simulators, it does not naturally address the issues related to the neuromorphic hardware implementations, such as AER translations, or model neuron placement on the VLSI array of silicon neurons. In PyNN these tasks are relegated to custom external software modules that are then imported into PyNN.

An attempt to provide developers with a unified hardware abstraction layer for integrating neuromorphic platforms in PyNN was the one of PyHAL project (Brüderle et al., 2007, 2009). The PyHAL software was intended to manage all the operations needed to execute the neural network simulations on the hardware, such as parameter translations, and perform the necessary checks to account for the hardware restrictions (e.g., in terms of number of neurons available or precision of model parameters such as time constants). This project was initially developed for the Heidelberg's accelerated neural network hardware but has not been pursued further^5^. Indeed, because of its “monolithic” structure, PyHAL is functionally equivalent to the dedicated software interfaces, e.g., developed for the SpiNNaker hardware. Rather than following this monolithic approach, PyNCS offers a more dynamic platform in which hardware devices with their corresponding low level driver modules are separated and can be loaded at runtime. The aim of this design philosophy is to encourage code portability, since modules can be shared within the community. At the cost of a small overhead due to the construction of the API layer, PyNCS leads to a simpler, more efficient and shareable code since single driver modules can be independently optimized and easily included in the ecosystem.

Regarding its front-end, PyNCS shares many similarities with existing solutions, offering a minimal, though complete, set of functionalities for neural network definition. Even more advanced languages such as IBM's Corelet language, which implements a Neuron class, a Core class and a Connector class, and uses these classes to define high-level Corelets, can be essentially mapped into the PyNCS front-end. The Neuron class contains all the properties of the neuron model, while in PyNCS the equivalent information is contained in the NHML files. The Core class represents a neurosynaptic core of 256 neurons with 256 × 256 synapses, and can be represented in PyNCS using Population and Connection objects. The Connector class defines the communication between cores and neurons, similarly to the Mapping class in PyNCS. Nonetheless, the Corelet language is a very powerful tool for approaching a novel programming paradigm but it is directly tied to IBM's specific hardware platform and cannot be used in conjunction with different neuromorphic platforms. In this sense, its nature profoundly differs from the one of PyNCS, which instead has been conceived as a sophisticated software infrastructure granting access to the widest possible range of spiking neuromorphic hardware.

We thus conclude that PyNCS is the first software ecosystem of its kind. As we have shown, a layer of APIs wrappers is used for the inclusion of existing communication or configuration software. In addition, we introduced a new mark-up language for providing a formal, unified description of neuromorphic devices, the NHML, and made extensive use of text description files for multi-chip setups written in XML as well. While text files and code wrappers cannot entirely eliminate dedicated coding efforts, the unique design philosophy of PyNCS reduces and streamlines these efforts. To achieve this, the software takes full advantage of Python built-ins and a novel hardware description language. It aims at promoting modularity of the hardware through modularity of the code, code re-use and coding simplicity by using common programming techniques which should be familiar to any moderately skilled Python developer, such as class-inheritance for the implementation of the APIs and context-managers for flexible interaction with the hardware at runtime.

A key functionality that is essential to harness the advantages of real-time hardware is continuous, online interaction with the system at runtime. Taking this into consideration, the design of PyNCS is not restricted to simulation-oriented hardware systems running in batch mode, instead it provides access to all the resources independently in a continuous manner. This is possible because the communication and configuration APIs are in fact independent thanks to the modularity of PyNCS and so, for example, hardware parameters can be changed while the system is running. On the other hand, real-time monitoring of spiking activity can be computationally intensive and could pose serious scalability issues that we address in the next section. Because of its continuous interaction capabilities, PyNCS allows one to close the loop between the neural network emulation on hardware and the world, while also including batch-processing capabilities, similar to PyNN, for compatibility with existing platforms.

### 5.2. Performance issues

The memory available to PyNCS can become a limiting factor for large-scale experiments. Memory is used for storing address-events and connection tables. In practice, we find that PyNCS can reasonably deal with about 10^8^ connections, and 10^5^ neurons. In terms of performance, for a virtual device comprising 32768 neurons, a laptop PC (Intel Core i7-2620M, 2.7 GHz, 4GB of RAM, Python 2.7.6) is able to generate, stimulate and monitor events at a rate of about 350,000 events per second. The processing time mostly consists in the filtering and sorting of the events and the transformation of physical addresses into logical addresses. A large portion of these operations are carried out using standard numerical libraries made available through the numpy module. As a result, the performance of PyNCS is in most cases not limited by the interpreted nature of Python. One way to overcome memory limitation in the future is to parallelize PyNCS using a message passing interface (Gropp et al., 1999).

### 5.3. A neuromorphic microkernel in python

The PyNCS software shares similarities with the kernel of a traditional operating system architecture.

In computers, a kernel is a bridge between applications using abstract resources and the actual data processing done at the hardware level through the management of the system's resources. Its function is to implement a collection of facilities of “universal applicability” and “absolute reliability” by which an arbitrary set of operating system facilities and policies can be conveniently, flexibly, efficiently, and reliably constructed (Wulf et al., 1974). In the framework of neuromorphic systems, the “operating system facilities” take the form of functional networks, e.g., FSM, that are constructed in the user-defined scripts and use the PyNCS front-end or “facilities” for configuring and access the hardware resources. As in the case of kernels, where different processors, interfaces, I/O devices, are typically supported through the specific drivers (or servers as in microlithic kernels, e.g., the Windows NT kernel), the neuromorphic kernel should manage the configuration of the different neuromorphic devices and mediate the communication between them. We proposed the definition of a communication and configuration infrastructure and the introduction of a set of specifications for the description of the low-level functionality as one possible strategy to fulfill this purpose. The setup files and NHML chip files represent the basis on which the PyNCS ecosystem operates. Based on a widely adopted digital communication framework, the AER (Boahen, 1998), and on a small set of basic assumptions they implement the hardware abstraction layer which enables the communication between the different hardware resources and the management from the front-end.

While existing solutions also offer similar functionalities, i.e., they implement a collection of facilities to properly configure the hardware, they are no longer appropriate in light of the recent developments in the growing community of neuromorphic engineering. An increasing number of reprogrammable, digital platforms are commercially available and can be used to interface spiking multi-neuron chips and sensors in a reliable manner. We witness the emergence of a trade of neuromorphic devices which requires the software ecosystem to be rapidly re-configurable with minimal coding effort and with the possibility for specific parts of it to be finely tuned without unintended side effects at the system level. Interestingly, this phenomenon is analogous to the introduction of *microkernels* in the computer industry, developed around the 80's as a response to the changes in the computer world^6^.

PyNCS is currently being tested on different electronic platforms and collaboratively improved by several research groups around the world. Further development of a complete kernel-like framework for neuromorphic devices should facilitate the development of cognitive systems and the assessment of their capabilities.

### 5.4. Improvements, limitations and outlook

The PyNCS software aims at creating a novel framework for developing software interfaces for neuromorphic platforms. To create such framework, we started with some assumptions that could already cover a broad range of neuromorphic platforms proposed in the literature. Certain aspects of this software call for further development but they will not affect the main design philosophy on which PyNCS has been conceived. The aim of PyNCS is not to propose one particular solution to neural network definition, mapping, placement or calibration but rather to offer a flexible and unified framework in which such routines can be included. Here we provide a list of modules that could be integrated in future releases.

One useful feature that PyNCS can easily support is real-time visualization of spiking data and real-time control of the neuromorphic chips through dedicated Graphical User Interfaces (GUIs). We developed and used preliminary versions of such modules, constructed as clients continuously gathering spiking data from servers, which in turn received data from the drivers. Importantly, these GUIs can be used not only for data visualization but also for real-time interaction with the system. For example, in an experimental version of the software, the user can send teacher signals to chips equipped with plastic synapses, thus realizing supervised online learning. However, since the clients heavily depend on the communication API used, these modules are not yet included in the mainstream version of PyNCS. An implementation of an API-independent module for real-time visualization and control is one of the most urgent targets of future versions of PyNCS.

In the examples that we show in this paper, neurons are composed of a spike-emitting block (the *soma*) and a spike-receiving block (the *synapse* array), each with their parameters shared within the block. Thus, these elements are dimension-less, i.e., PyNCS works under the point-neuron assumption. Instead, architectures involving multi-compartmental neurons can have more complicated parameter dependencies as well as addressing schemes. One possibility to include multi-compartmental neurons as well as dendritic computation and any other model that deviates from the simple soma-synapse representation could be to include in the NHML chip description more sophisticated block representations as required by the specific models. If verified, this possibility would be the ideal demonstration of the flexibility of the NHML code and we are actively seeking ways to explore this issue.

In the development of PyNCS, we deliberately omitted two very important elements for the construction of a neuromorphic setup: placement routines and calibration routines. Though these routines are in some cases fundamental for the realization of the system, their implementation would not have added more value in the demonstration of the principles behind our software. As mentioned already in Section 3, these routines can be included under the configuration API. For example, when the run command is executed, PyNCS would *compile* the neural network definition using the methods implemented in a ConfAPI.Placement module and a ConfAPI.Calibration module. These routines could also integrate automated checks to raise warnings or exceptions in case resources are unavailable. For example, an exception could be raised when one tries to define a neuronal population consisting of more neurons than the ones actually available in the devices. Though these modules are not yet part of PyNCS, we already used its core elements (i.e., the high-level neural network interface, the address translation methods and the access to hardware parameters) to run calibration routines for parameter optimization, demonstrating the possibility to effectively realize such improvements (Neftci et al., 2010, 2011, 2012; Sheik et al., 2011). In particular, calibration of analog circuits needs means to access analog variables. PyNCS does not yet support the direct control of measurement instruments, which could be included as a module under the communication API, however a more convenient possibility is to employ circuits for converting internal analog variables, e.g., synaptic currents or membrane potentials, into AER signals and use these to infer their real value, a technique which is commonly used for rapid calibration of analog circuits (Serrano-Gotarredona et al., 2007; Yang et al., 2012).

## 6. Conclusions

We presented a modular and expandable platform-independent Python-based framework to control spike-based neuromorphic systems based on the AER communication. We showed how these Python software tools can be used to configure neuromorphic hardware setups for emulating spiking neural networks. By taking advantage of the flexibility of Python, we designed PyNCS as a collection of open source APIs that grant access to all the low-level functionalities of the neuromorphic hardware while also allowing the integration to high-level front-ends for neural-network definition. We proposed to adopt a general scheme based on specification files for the electronic hardware, in the form of XML code, to facilitate the integration of custom hardware into existing systems. We demonstrated the use of our software with experiments which included a spiking neuromorphic vision sensor, the remote transmission of spiking data in real-time via TCP protocol and recurrent neural networks implementing finite-state machines for general purpose computation. Moreover, we reported previous work that made use of PyNCS for neural network models operating on precisely timed spiking input. We believe that the software tools we presented will encourage the integration of the diverse range of neuromorphic systems available today with the consequence of a tighter exchange of expertise among the groups in the community, a wider adoption of neuromorphic platforms for both simulation and beyond-simulation purposes, and a more application-aware development of new hardware resources as future emerging technologies.

### Conflict of interest statement

The authors declare that the research was conducted in the absence of any commercial or financial relationships that could be construed as a potential conflict of interest.

## Footnotes

^1^Proceedings of the IEEE, Vol. 102, No. 5, May 2014

^2^PyGame is available at http://www.pygame.org. The script to generate the sliding bars is available at https://github.com/inincs/stefanini_etal_pyncs

^3^The syntax is inspired by the Brian Simulator (Goodman and Brette, 2008).

^4^See also the pyNN.SpiNNaker website at http://spinnaker.cs.man.ac.uk/docs/spinnaker_package/index.html

^5^The Heidelberg team substituted the PyHAL hardware abstraction layer by a dedicated and private PyNN module which only supports the team's hardware so far. See http://neuralensemble.org/trac/PyNN/wiki/NeuromorphicHardwareFAQ.

^6^From Wikipedia: http://en.wikipedia.org/wiki/Microkernel
