# Supplementary material for: PyNCS: a microkernel for high-level definition and configuration of neuromorphic electronic systems
Source: Front Neuroinform. 2014 Aug 29;8:73. doi: 10.3389/fninf.2014.00073 (PMC4152885; doi:10.3389/fninf.2014.00073)
Supplement: Supplementary file 1 [file DataSheet1.PDF]

Frontiers in Neuroinformatics

Research Article
10 April 2014

Supplementary material: PyNCS: a microkernel for high-level definition and configuration of neuromorphic electronic systems

Fabio Stefanini [2,†], Emre Neftci [1,*,†], Sadique Sheik [2,†], Giacomo Indiveri [2]

[1] *Institute for Neural Computation, UCSD, La Jolla, CA, USA*
[2] *Institute of Neuroinformatics, University of Zurich and ETH Zurich, Zurich, Switzerland*
† *equal contributors*

Correspondence*:
Fabio Stefanini
Institute for Neuroinformatics, Winterthurerstrasse 190, 8057 Zurich, Switzerland,
fabio.stefanini@ini.phys.ethz.ch

Research Topic

ADDRESS REPRESENTATIONS AND MONITORING TOOLS

A central feature in PyNCS concerns address translation. In the most basic Address Event Representation (AER) system, digital addresses are associated to spiking neurons in neuromorphic devices by local *encoders*, generating a unique digital string whenever a neuron spikes which corresponds to that neuron, and to synapses by *decoders*, which direct digital pulses to the synapse which correspond to the received address. These digital addresses (unsigned integers) are referred to as *physical* addresses, because they are physically associated to the corresponding spike sources and targets. To facilitate operations with these addresses, PyNCS associates to each addressable unit two more abstract representations, with a one-to-one correspondence between them and the physical addresses: a “Human Readable (HR) address”, which is a tuple of unsigned integers specifying the coordinates of the neuron or synapse (e.g. neuron’s x coordinate, neurons’s y coordinate, synapse) and a positive, real-numbered “logical” address. HR addresses are useful for specifying the network topology but can be difficult to use because of their multiple dimensions, especially during visualization. To simplify the of multidimensional addresses, PyNCS makes use of the “logical” addresses. The integer part of the logical address specifies the collapsed address of the soma and the fractional part of the number specifies the (possibly multidimensional) synapse according to:

$$a_l = \sum_{i=1}^{n} 2^{\sum_{j=0}^{n-1} N_j} \cdot X_i + \sum_{i=1}^{m} 2^{-\sum_{j=1}^{m} M_j} \cdot S_i,$$

where a_l is the logical address, N_i is the number of bits associated to dimension i ($N_0 = 0$), M_i is the number of bits associated to the synaptic dimension i. For example, on a multineuron chip having one neuron per node of a 64 dimensional grid, with 4 synapses per neuron, a synapse with HR coordinate (23, 12, 2) has logical address $791.5 = 12 \cdot 2^6 + 23 + 2 \cdot 2^{-2}$ and a synapse on a neighboring neuron (24, 13, 2) has address 792.5. When sorted, the addresses are ordered according to the first coordinate, which is convenient for spike train visualization and analysis. The three types of addresses described above:

integers, tuples and floating point numbers are immutable in Python, enabling them to be used as hash table keys (dictionaries). This feature is used for fast translation between the three types.

To analyze and plot spike trains PyNCS makes use of `SpikeLists`. SpikeLists have a multitude of functions for computing the basic statistics of spike trains and plotting. It is an adapted version of the SpikeList class found in the NeuroTools module (`http://www.neuralensemble.org/NeuroTools/`). PyNCS SpikeLists are therefore compatible with NeuroTools for extensive spike train analysis. The SpikeMonitor is a SpikeList container that allows to seamlessly monitor the activity of a population neurons. At the end of “run()”, each monitor is populated by the events whose addresses match those of the monitored population.

CHIP AND SETUP DESCRIPTIONS FILES

Chip description files contain the specific informations required to configure and communicate with each neuromorphic chip. They are written in a newly defined mark-up language named Neuromorphic Hardware Mark-up Language (NHML). Here we provide an example of an NHML that describes the neurons, synapses and parameters available in a neuromorphic chip that consists of a two-dimensional sheet of 2048 neurons, with 4096 excitatory and 2048 inhibitory AER synapses.

```
<chip chipclass="IF2DWTA">

    <addressSpecification type="aerIn">
        <dim id="x" type="soma">
            <range>range(64)</range>
            <description/>
            <decoder>X&gt;&gt;1</decoder>
        </dim>
        <dim id="y" type="soma">
            <range>range(32)</range>
            <description/>
            <decoder>((Y-(Y%3))&lt;&lt;1)/3+(X&amp;1)</decoder>
        </dim>
        <dim id="s" type="synapse">
            <range>[0, 1, 2]</range>
            <description/>
            <decoder>Y%3</decoder>
        </dim>
        <pin id="X">
            <decoder>2*x+(y&amp;1)</decoder>
        </pin>
        <pin id="Y">
            <decoder>s+3*(y&gt;&gt;1)</decoder>
        </pin>
        <pinlayout>Y0 Y1 Y2 Y3 Y4 Y5 X6 X5 X4 X3 X2 X1 X0</pinlayout>
    </addressSpecification>
    <!-- aerOut ADDRESS SPECIFICATION HERE... -->

    <parameters>
        <parameter
            Count="41.0"
            SimulationValue="0.0"
            Description=""
            Pin="51.0"
            BiasType="dac"
            PADtype=""
```

```
            FET=""
            Range="[0.05,␣0.2]"
            Pad=""
            CircuitBlock="SYNAEREXC"
            Channel="78.0"
            SignalName="nsynaerexcplswdt"
            />
        <!-- OTHER PARAMETERS HERE -->
    </parameters>

    <neuron id="excitatory">
        <soma type="SOMA" id="general">
            <dim id="y" range="range(32)"/>
            <dim id="x" range="range(64)"/>
            <parameter id="refractory" SignalName="nrf"/>
            <!-- OTHER PARAMETERS HERE -->
        </soma>
        <synapse type="EXCITATORY␣SYNAPSE" id="excitatory1">
            <dim id="s" range="[2]"/>
            <parameter id="threshold" SignalName="nsynaerexcth1"/>
            <!-- OTHER PARAMETERS HERE... -->
        </synapse>
        <!-- OTHER SYNAPSES, E.G., INHIBITORY, HERE... -->
    </neuron>

    <!-- OTHER NEURONS HERE... -->

</chip>
```

An outline of a 4 bit setup type description file is shown below. The file lists the available channels in the setup together with their corresponding reserved chunks of addresses.

```
<?xml version="1.0" ?>
<setuptype version="0.1">
    <channelAddressing bits="[17,␣18,␣19,␣20]" name="default" type="monitor"/>
    <channelAddressing bits="[17,␣18,␣19,␣20]" name="default" type="sequencer"/>
    <slot id="0">
        <aerMon in="[8]" out="[0]"/>
        <aerSeq in="[8]" out="[0]"/>
    </slot>
    <!--> Here follow other 2^3-1 slots... <-->
</setuptype>
```

In the following code the actual configuration of a neuromorphic setup is outlined. It is based on the 4 bit setup type listed in the previous code. The code defining the content of the setup is provided to PyNCS in the form of an eXtensible Mark-up Language (XML) file called *setup file*.

```
<?xml version="1.0" ?>
<!DOCTYPE document SYSTEM "setup.dtd">

<!--> A multi-chip configuration based on a 4-bit setup. <-->
<setup version="1.0">

    <chip chipfile="my_chip.nhml" id="chip" slot="1">
        <configurator module='pyMyConf.api'>
            <parameter name='host'>some.host.com</parameter>
            <parameter name='board'>208</parameter>
```

```
        </configurator>
    </chip>

    <!--> Here other <chip> objects... <-->

    <virtualchip chipfile='my_reteina.nhml' id='retina' slot='5' />
    <defaultchip chipfile="default.nhml" />

    <communicator module='pyMyCom.api.com_client'>
        <parameter name='host'>some.com.host.com</parameter>
        <parameter name='fps'>25</parameter>
        <!--> Other ComAPI-related parameters here... <-->
    </communicator>

    <mapper module='pyMyMap.api.conf_client'>
        <parameter name='host'>some.map.host.com</parameter>
        <parameter name='version'>3.0</parameter>
        <!--> Other MapAPI-related parameters here... <-->
    </mapper>

</setup>
```

The two XML files describing the hardware setup provide all the necessary information for PyNCS and its internal modules to communicate with it and configure it. This information is parsed from the files on initialization of the `NeuroSetup` as follows.

```
# SETUP DEFINITION
setup = pyNCS.NeuroSetup('setuptype.xml','setupfile.xml')
```

More code and examples are available at `https://github.com/inincs`.

DETAILS TO INTERFACING A SPIKING NEUROMORPHIC CHIP WITH A SILICON RETINA

The visual stimulus has been produced using the PyGame interface (`http://www.pygame.org`). Two horizontal light bars of different size move and 10% difference in brightness slide on a dark background in two opposite directions. The canvas is a torus, such that when a bar hits the border it disappears and reappears on the other side of the canvas. The code is also available at `http://ncs.ethz.ch/projects/neurop/`.

```
import pygame, sys
from pygame.locals import *

pygame.init()
fpsClock = pygame.time.Clock()

win_width, win_height = 640, 480
windowSurfaceObj = pygame.display.set_mode((win_width, win_height))
pygame.display.set_caption('Sliding_bar')

grayColor = pygame.Color(0, 0, 0)
bars_contrast = 0.1 # luminance contrast between bars
bar1Color = pygame.Color(255, 255, 255)
bar2Color = pygame.Color(255-int(bars_contrast*255),
                         255-int(bars_contrast*255),
```

```
                         255-int(bars_contrast*255))

pos_left1 = 10 # initial position of the bar1
pos_top1 = 10
delta_left1 = 5
delta_top1 = 5
width1 = 100
height1 = 20

pos_left2 = -10 # initial position of the bar
pos_top2 = 10
delta_left2 = -5
delta_top2 = -5
width2 = 50
height2 = 20

while True:
    windowSurfaceObj.fill(grayColor)

    pygame.draw.rect(windowSurfaceObj,
                     bar1Color,
                     (pos_left1, pos_top1, width1, height1))

    pygame.draw.rect(windowSurfaceObj,
                     bar2Color,
                     (pos_left2, pos_top2, width2, height2))

    pos_top1 += delta_top1
    pos_left1 += delta_left1
    pos_left1 %= win_width - width1
    pos_top1 %= win_height - height1

    pos_top2 += delta_top2
    pos_left2 += delta_left2
    pos_left2 %= win_width - width2
    pos_top2 %= win_height - height2

    for event in pygame.event.get():
        if event.type == QUIT:
            pygame.quit()
            sys.exit()

    pygame.display.update()
    fpsClock.tick(20)
```

The following code listing shows an example of PyNCS usage to configure and monitor a neuromorphic setup consisting of a silicon retina and a multi-neuron chip.

```
from pyNCS import Population, Connection, NeuroSetup, monitors
import contextlib
from visual_stimulus import init_vstim, term_vstim

# Initialize setup
setup = pyNCS.NeuroSetup('setupfiles/4bit_setuptype.xml', 'setupfiles/ssm_setup.xml
    ')
set_default_biases(setup)
```

```
#POPULATIONS
p_ret = Population('Retina')
p_wta = Population('Exc.')
p_wta_inh = Population('Inh.')

p_ret.populate_all(setup, 'retina', 'pixelon')
p_wta.populate_all(setup, 'ifslwta0', 'excitatory')
p_wta_inh.populate_all(setup, 'ifslwta0', 'inhibitory')

#CONNECTIONS
C = [Connection(p_ret[i:(2**12):64], p_wta[(2*i):(2*(i+1))], 'excitatory0',
     fashion = 'random_all2all',
     fashion_kwargs = {'p' : 0.5}) for i in range(62)]

mons = setup.monitors.create([p_ret,p_wta,p_wta_inh])

#VISUAL CONTEXT
@contextlib.contextmanager
def visual_exp_cm():
    init_vstim()
    yield #sequencing and monitoring begins here
    term_vstim()

#RUN
setup.run(duration = 10000, context_manager = visual_exp_cm)

#PLOT
monitors.RasterPlot(mons)
```

The setup files used in this example are identical to the ones used in the article. After the setup object is created, the populations associated to the retina (here we take only neurons of type `pixelon`, i.e., those that are associated with increase in contrast), the excitatory neurons of the Soft Winner–Take–All (sWTA) and its inhibitory neurons are defined. The neuron types, their numbers and associated synapses are described in the chip files. Each column in the retina projects to two neighboring neurons in the multi-neuron chip. The probability of a connection to exist between each pair of neurons is 0.5. The `pyNCS.monitors` module is directly accessed via the setup object, and allows for the creation of spike monitors associated to each population. Using a context manager, the function that initiates the visual scene can be passed to the API call and the low-level driver. The API module calls this function immediately before the monitoring is initiated. This allows the visual scene to be almost perfectly synchronized with the monitoring events. Finally, the experiment is run by calling `setup.run()` and raster plots of the monitored populations are created.

DETAILS TO SSM GENERATION

The following code listing shows the script generating the Soft State Machines (SSMs). The SSM is configured using the state transition table, which describes all the possible state transitions conditioned on input symbols. Here, the state machine is specified in `fsm` and the sWTA couplings implementing the persistent activity already exist (i.e. they are hard-wired). First, the populations associated to the SSM states, input symbols and all possible transitions are created. Input symbols are provided by an external source of address-events, generated on a desktop PC, with 16 neurons per input symbol. The state populations consist of 32 neurons, configured such that their activity can persist in the absence of inputs. By construction, each state population is in competition with all the other state populations mediated by a common inhibitory population.

Each possible transition between two states is mediated by a 16 neuron population in the “transition sWTA”, and is triggered by an input symbol. For each possible transition, there is a population in the transition sWTA which is activated by that symbol and the population of the previous state through excitatory connections. The transition population is configured such that it can activate only if the two efferent populations are simultaneously active. The output of each transition population is mapped on to the target state populations.
